# Prospective Multicenter Study of Community-Associated Skin and Skin Structure Infections due to Methicillin-Resistant *Staphylococcus aureus* in Buenos Aires, Argentina

**DOI:** 10.1371/journal.pone.0078303

**Published:** 2013-11-20

**Authors:** María José López Furst, Lautaro de Vedia, Silvina Fernández, Noella Gardella, María Cristina Ganaha, Sergio Prieto, Edith Carbone, Nicolás Lista, Flavio Rotryng, Graciana I. Morera, Marta Mollerach, Martín E. Stryjewski, Grupo de Estudio de Infecciones de Piel y Estructuras Relacionadas por Staphylococcus aureus meticilino-resistente de la Comunidad, Sociedad Argentina de Infectología

**Affiliations:** 1 Unidad de Infectología, Sanatorio Municipal Dr. Julio Méndez, Ciudad Autónoma de Buenos Aires, Argentina; 2 Departamento de Atención Intensiva al Paciente Infectológico Crítico, Hospital Francisco J. Muñiz, Ciudad Autónoma de Buenos Aires, Argentina; 3 Cátedra de Microbiología, Facultad de Farmacia y Bioquímica, Universidad de Buenos Aires, Ciudad Autónoma de Buenos Aires, Argentina; 4 Sala de Infectología, Hospital Vicente López y Planes, Gral. Rodríguez, Buenos Aires, Argentina; 5 Servicio de Enfermedades Infecciosas, Hospital Nuestra Señora de Luján, Luján, Buenos Aires, Argentina; 6 Departamento de Clínica Médica, Hospital Aeronáutico Central, Ciudad Autónoma de Buenos Aires, Argentina; 7 Servicio de Infectología, Hospital Universidad Abierta Interamericana, Ciudad Autónoma de Buenos Aires, Argentina; 8 Sección de Infectología, Hospital Dr. Jose Cullen, Santa Fe, Argentina; 9 Sección de Infectología, Centro de Educación Médica e Investigaciones Clínicas “Norberto Quirno” (CEMIC), Ciudad Autónoma de Buenos Aires, Argentina; University Hospital Münster, Germany

## Abstract

**Background:**

Community-associated methicillin-resistant *Staphylococcus aureus* (CA-MRSA) is now the most common cause of skin and skin structure infections (SSSI) in several world regions. In Argentina prospective, multicenter clinical studies have only been conducted in pediatric populations.

**Objective:**

Primary: describe the prevalence, clinical and demographic characteristics of adult patients with community acquired SSSI due to MRSA; secondary: molecular evaluation of CA-MRSA strains. Patients with MRSA were compared to those without MRSA.

**Materials and Methods:**

Prospective, observational, multicenter, epidemiologic study, with molecular analysis, conducted at 19 sites in Argentina (18 in Buenos Aires) between March 2010 and October 2011. Patients were included if they were ≥14 years, were diagnosed with SSSI, a culture was obtained, and there had no significant healthcare contact identified. A logistic regression model was used to identify factors associated with CA-MRSA. Pulse field types, SCC*mec*, and PVL status were also determined.

**Results:**

A total of 311 patients were included. CA-MRSA was isolated in 70% (218/311) of patients. Clinical variables independently associated with CA-MRSA were: presence of purulent lesion (OR 3.29; 95%CI 1.67, 6.49) and age <50 years (OR 2.39; 95%CI 1.22, 4.70). The vast majority of CA-MRSA strains causing SSSI carried PVL genes (95%) and were SCC*mec* type IV. The sequence type CA-MRSA ST30 spa t019 was the predominant clone.

**Conclusions:**

CA-MRSA is now the most common cause of SSSI in our adult patients without healthcare contact. ST30, SCCmec IV, PVL+, spa t019 is the predominant clone in Buenos Aires, Argentina.

## Introduction

Community-associated methicillin-resistant *Staphylococcus aureus* (CA-MRSA) has emerged in different world regions [1] including Latin America [2], [3] as a major cause of acute bacterial skin and skin structure infections (SSSI). Different from hospital-acquired MRSA, CA-MRSA usually is *mec* type IV, carries genes for Panton-Valentine leukocidin (PVL) and is susceptible to several non ß-lactam antibiotics [4].

In Argentina prospective, multicenter, studies with molecular evaluation of community MRSA in patients with SSSI have only been conducted in pediatric populations [5], [6]. However, despite the observation that the clinical characteristics of SSSI in adults appear similar to those of children the prevalence of CA-MRSA in adolescents and adults presenting with SSSI infection have not been determined.

The epidemic of CA-MRSA is evolving [7]. A specific clone that is initially propagating and causing CA-MRSA infections can be displaced by a more successfully one. For example in the US the initially reported CA-MRSA clone USA 400 was subsequently replaced by the USA 300 which became the most common cause of SSSI [1], [8]. In Argentina previous studies have identified ST5, SCCmec IV, spa type 311 as the predominant CA-MRSA clone causing infections [6] and colonizing children [9]. Microbiologically based studies have suggested the same clonal predominance of CA-MRSA among Argentinean adults [10]. Whether this clone of CA-MRSA is still dominant or has been replaced in adult patient with SSSI in our country needs to be determined. The current study was conducted to establish the prevalence, clinical and molecular characteristics of CA-MRSA in adolescents and adults with SSSI in Argentina.

## Materials and Methods

A prospective, observational, multicenter study was conducted in a total 19 centers (18 in Buenos Aires state and city, and 1 in Santa Fe state) between March 2010 and October 2011. The primary objective was to determine prevalence, clinical and demographic characteristics in patients with SSSI due to CA-MRSA. Secondary objective was to perform a molecular analysis of CA-MRSA strains.

Patients were included if they had ≥14 years old, presented with SSSI and had a culture obtained. Patients were excluded if they had any of the following contacts with the healthcare system within the last 12 months: hospitalization, chronic care (e.g. nursing home), catheter placement, dialysis or surgery.

The information was collected using an electronic clinical report form (eCRF). Patients were followed for at least one visit after the end of antimicrobial therapy. Clinical outcomes were defined as follows: a) *cure*, resolution of signs and symptoms of infection to the point at which no further antibiotics or procedures were deemed necessary, b) *failure*, need for a different antibiotic based on lack of clinical response, worsening of signs or symptoms, and/or need for an additional surgical procedure after end of therapy, and c) *indeterminate*, unable to assess clinical outcome (e.g. missing follow up). Clinical outcomes of cure and failure were captured during medical visits conducted for regular care (not a fixed time point). Predisposing factors for SSSI and CA-MRSA infections were selected from the literature. Data was analyzed utilizing SAS 9.3 (Cary, North Carolina). Patients with CA-MRSA were compared to those without CA-MRSA (culture negative or pathogens other than CA-MRSA) using Chi square or Fisher's exact test (categorical variables) and t-test or Wilcoxon rank sum (continous variables), as appropriate. A logistic regression model (backward stepwise) was conducted to identify clinical variables associated with CA-MRSA infection. Clinically relevant variables with p values p≤0,05 in the univariate analysis were included in the model. The information of the study was kept confidential until the database was anonymized.

Pathogen identification and antibiotic susceptibility was obtained from each institutional microbiology laboratory. MRSA isolates were sent to a reference laboratory (Cátedra de Microbiología, Facultad de Farmacia y Bioquímica, Universidad de Buenos Aires). Resistance to methicillin was confirmed through PCR amplification of *mec*A gene. *S. aureus* ATCC strains 43300 and 29213 were used as positive and negative controls, respectively. SCC*mec* types were identified analyzing *mec* and *ccr* elements [11]. Presence Panton-Valentine leucocidin (PVL) genes (*lukS/F-PV*) were also determined [12].

Pulse field gel electrophoresis (PFGE) was performed as described by Chung [13]. PFGE also included locally circulating clones (e.g. clone CAA, pulse field type A, ST5, *spa*t311, SCC*mec*IV, PVL+) and one strain characterized as pulse field type C, ST30, *spa*t019, SCC*mec*IV, PVL+ [14]. The analysis of PGFE profiles was carried out by visual inspection. A dendrogram (Treecon 1.3b) was built applying unweighted pair-group method clustering analysis (UPGMA) algorithm and Dice coefficient. Isolates with similar band pattern (≥85%) were considered to belong to the same pulse field type. Profiles observed were named according to their similarity to control pulse field types (A and C). Genotype analysis of isolates was also performed using *spa* typing [15]. Data obtained was analyzed using a reference website: http://www.ridom.de/spaserver (last accessed on November 20^th^ 2012). A representative proportion of isolates from each pulse fieldtype were studied using multi locus sequence typing (MLST) (http://saureus.mlst.net/; last accessed on November 20^th^ 2012)

The study was approved by Institutional Review Boards (IRB) from participating institutions. In all cases the adult participants gave written or verbal consent as required by the IRBs. In accord with local IRB instructions the following participating sites required written informed consent (ICF): Hospital Dr. Josè María Cullen, Hospital Español de Buenos Aires, Hospital Bernardo Houssay de Vicente López, Htal. F. Santojanni, Policlínico Central de La Matanza (for patients <18 years old) and Sanatorio Otamendi. At the above sites written informed consent forms for patients <18 years old were obtained from parents or legal guardians on the behalf of the participant minors/children. Given the observational nature of the study and the anonymization of data the remaining IRBs from participating institutions did not required written ICF (regardless of patient's age). The IRBs from the following participating sites approved the study and required only verbal consent without need of documentation: Centro de Educación Médica e Investigaciones Clínicas (CEMIC), Htal. Aeronáutico Central, Htal. Español de Buenos Aires, Htal. Evita Pueblo, Htal. Juan A. Fernández, IPER, Htal. Nuestra Señora de Luján, Htal. Privado de la Comunidad, Htal. Tornú, Htal. Universidad Abierta Interamericana, Htal. Velez Sarfield, Htal. IGA Vicente López, Policlínico Central de La Matanza (for ≥18 years old), Sanatorio Municipal Dr. Julio Méndez, Sanatorio Tandil. The verbal consent for patients <18 years old were obtained from parents or legal guardians on the behalf of the minors/children participants. There was no need for IRB approval at Hospital Británico de Buenos Aires (study previously discussed with the site IRB and in compliance with their policies for observational studies with anonymized data; verbal waiver obtained from the IRB board members).

## Results

A total of 311 patients were enrolled during the study period in 19 centers. The majority of patients were male (60%) and the mean age was 38,8 (±18,1) years old (Table 1). A history of previous furuncles was the most predisposing factor for SSSI (36%). Almost 70% of patients had an identifiable predisposing factor for CA-MRSA. Among such factors the receipt of antibiotics within the last 12 months was the most common. Abscesses and furuncles were the most frequent SSSIs accounting for 70% of the cases. A vast majority of patients had purulent lesions and one third presented with fever. Almost 90% (271/311) of patients had positive cultures. Among subjects with positive cultures C-MRSA was obtained in 80,4% followed by methicillin-susceptible *S. aureus* (MSSA) which was isolated in 11,1% of patients, respectively(Table 2).

**Table 1:** Clinical and demographic characteristics in patients with skin and skin structure infections due to community-associated MRSA.

Variables		Total N = 311n/N (%)	CA- MRSA N = 218n/N (%)	No MRSA N = 93n/N (%)	p*
**Demographic characterstics**	Gender male	187/311 (60,1%)	133/218 (61%)	54/93 (58,1%)	0,63
	Age in years, mean (±SD)	38,8 (±18,1)	36,1 (±16,7)	45,3 (±19,7)	0,0001
	<50 years old	225/307(73,6%)	172/215 (80,0%)	53/92 (57,6%)	<0,0001
	BMI, mean (± SD)	26,5 (±5,7)	25,8 (±5,1)	28,2 (±6,6)	0,006
	BMI≤25	107/239 (44,8%)	82/166 (49,4%)	25/73 (34,2%)	0,03
**Predisposing factors for skin infections**	Total†	232/311 (74,6%)	160/218 (73,4%)	72/93 (77,4%)	–
	Furunculosis (history)	113/311 (36,3%)	93/218 (42,7%)	20/93 (21,5%)	0,0004
	Trauma	44/311 (14,2%)	29/218 (13,3%)	15/93 (16,1%)	0,51
	Diabetes	37/311 (11,2%)	23/218 (10,6%)	14/93 (15,1%)	0,26
	HIV	24/311 (7,7%)	20/218 (9,2%)	4/93 (4,3%)	0,14
	Peripheral vascular disease	22/311 (7,1%)	12/218 (5,5%)	10/93 (10,8%)	0,1
	Immunosuppressant therapy	22/311 (7,1%)	11/218 (5,1%)	11/93 (11,8%)	0,03
**Predisposing factors for CA-MRSA**	Total	217/311 (69,8%)	167/218 (73,6%)	50/93 (53,8%)	–
	Previous antibiotics last 12 months	152/311 (48,9%)	117/218 (53,7%)	35/93 (37,6%)	0,01
	Previous antibiotics last 30 days	121/311 (38,9%)	90/218 (41,3%)	31/93 (33,3%)	0,19
	House contacts with similar lesions	78/311 (25,1%)	64/218 (29,4%)	14/93 (15,1%)	0,008
	Contact sports	49/311 (15,8%)	37/218 (17,0%)	12/93 (12,9%)	0,37
**Nasal swab, MRSA positive** ‡		15/65 (23,1%)	15/50 (30,0%)	0/15 (0%)	0,01
**Type of lesion**					<0,0001
	Furuncle	111/311 (35,7%)	88/218 (40,4%)	23/93 (24,7%)	–
	Abscess	107/311 (34,4%)	91/218 (41,7%)	16/93 (17,2%)	–
	Celullitis	77/311 (24,8%)	33/218 (15,1%)	44/93 (47,3%)	–
	Ulcer	9/311 (2,9%)	5/218 (2,3%)	4/93 (4,3%)	–
	Fasciitis	4/311 (1,3%)	0/218 (%)	4/93 (4,3%)	–
	Erisypela	2/311 (0,6%)	0/218 (0%)	2/93 (2,2%)	–
	Burn	1/311 (0,3%)	1/218 (0,5%)	0/93 (0%)	–
**Clinical characteristics**	Fever	102/311 (32,8%)	70/218 (32,1%)	32/93 (34,4%)	0,69
	Skin lesion multiple	93/311 (29,9%)	77/218 (35,3%)	16/93 (17,2%)	0,001
	Skin lesion purulent	236/311 (75,9%)	184/218 (84,4%)	52/93 (55,9%)	<0,0001
	Skin lesion necrotic	26/311 (8,4%)	13/218 (6,0%)	13/93 (13,4%)	0,02
	WBC>10×10^9^/L	78/126 (61,9%)	53/84 (63,1%)	25/42 (59,5%)	0,70
	Antibiotics within the last 72 hours	145/311 (46,7%)	104/218 (47,7%)	41/93 (44,1%)	0,56

MRSA denotes methicillin-resistant *Staphylococcus aureus*; CA-MRSA denotes community-associated MRSA; BMI, body mass index.

Data are displayed with n/N (%), except for continuous variables which are expressed by mean or median (standard deviation or interquartile range).

Predisposing factors for skin infections as well predisposing factors for Community MRSA displayed in this table were selected from medical literature. Comparisons were exploratory.

* Comparing patients with community-associated MRSA vs. those without community-associated MRSA.

† Including furunculosis as a predisposing factor.

‡ From the total of patients with nasal swabs.

**Table 2:** Microbiological results and MRSA susceptibilities in patients with skin and skin structure infections.

Variables		n/N (%)
**Positive culture**	Total	271/311 (87,1%)
	Monomicrobial	267/271 (98,5%)
**Most frequent pathogens** *	MRSA	218/271 (80,4%)
	MSSA	30/271 (11,1%)
	Coagulase negative staphylococci	5/271 (1,8%)
	*S.pyogenes*	3/271 (1,1%)
	*S. viridans*	4/271 (1,5%)
	*Streptococcus* group B, C, G	5/271 (1,8%)
	*P. aeruginosa*	3/271 (1,1%)
	Other	7/271 (2,6%)
**Culture of primary skin lesion**		311/311 (100%)
**Type of culture** †	Needle aspiration	265/311 (85,2%)
	Surgical sample	55/311 (17,7%)
**MRSA susceptibilities** ‡	Minocycline	141/141 (100%)
	Rifampin	184/186 (98,9%)
	TMP-SMX	207/210 (98,6%)
	Quinolones	175/185 (94,6%)
	Aminoglycosides	153/166 (92,2%)
	Clindamycin	188/211 (89,1%)
	Macrolides	172/200 (86,0%)

MRSA denotes methicillin-resistant *Staphylococcus aureus*; MSSA, methicillin-susceptible *Staphylococcus aureus*; TMP-SMX, trimethoprim-sulphametoxazole.

* From the total of patients with positive cultures; 275 pathogens were isolated from 271 patients; 4 patients had two pathogens isolated, respectively; other pathogens include *Proteus mirabilis* (n = 2), *Citrobacter spp* (n = 2), *Acinetobacter spp* (n = 1), E.coli (n = 1), *E. faecalis* (n = 1).

† A single patient may have more than one type of culture.

‡ From the total of isolates tested; susceptibilities were determined at each microbiology laboratory following their standards.

Patients infected with CA-MRSA (compared to those without CA-MRSA) were younger (36,1 vs. 45,3 years old; p = 0,0001) and had lower body mass index (BMI) (25,8 vs. 28,2; p = 0,006). Patients with C-MRSA more frequently gave a history of furuncles (42,7% vs. 21,5%; p = 0.0004), contacts with persons exhibiting similar skin lesions (29,4% vs. 15,1%; p = 0,008), and purulent (84% vs. 56%; p = <0.0001) or multiple lesions (35% vs. 17%; p = 0.005) (Table 1). Patients infected with CA-MRSA were more commonly found to have abscesses (41,7% vs. 17,2%) or furuncles (40,4% vs. 24,7%) and less frequently were diagnosed with cellulitis (15,1% vs. 47,3%) than patients without CA-MRSA (p<0,0001). Necrotic lesions were less common in patients with CA-MRSA (6% vs. 13,4%; p = 0,02).

**Figure 1:**
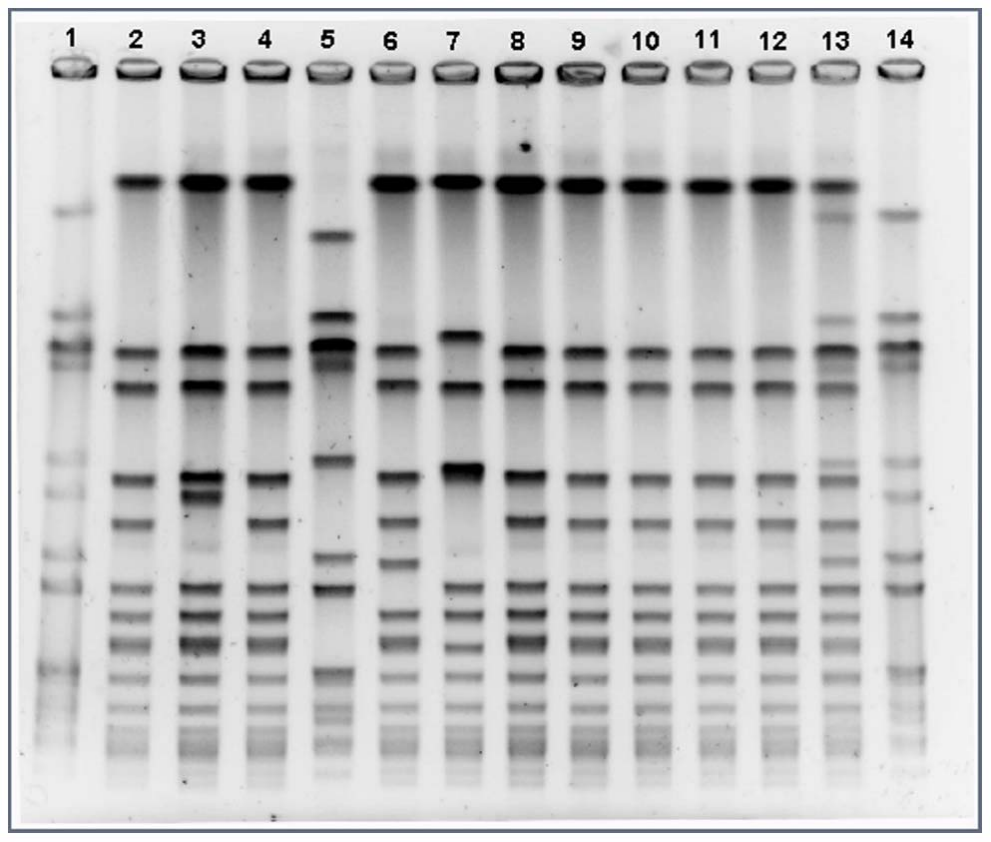
Pulse field patterns of representative community MRSA isolates in patients with skin and skin structure infections. Lane 1 and 14, pulse field type A clone (CAA); lane 2, control pulse field type C; lane 5, pulse field type A; lanes 13 and 7 other pulse field types; lanes 3, 4, 6 and 8–12 pulse field type C.

All CA-MRSA strains tested were susceptible to minocycline and more than 95% were susceptible to trimethoprim-sulfamethoxazole and rifampin. Resistance to clindamycin was detected in 11% and resistance to ≥2 non ß-lactam antibiotics was present in 12% of community MRSA strains, respectively. From the 146 MRSA isolates analyzed in the central laboratory methicillin resistance was confirmed in all and PVL genes were present in 94.5% of strains. SCC*mec* type IV was detected in 93.1% (136/146) of CA-MRSA isolates and only one strain had SCC*mec* type V. The predominant clone of CA-MRSA in patients with SSTI was characterized as pulse field type C, sequence type 30 (ST-30), *spa* t-019 (Table 3). The most common PFGE types identified are displayed in Figure 1.

**Table 3:** Molecular characteristics of community-associated MRSA in patients with skin and skin structure infections: pulse fieldtypes, sequence types, *mec* and *spa* types.

PFGE type	Isolates (n)	ST	SCC*mec* (n)	*spa* type (n)*
A	35	5	IV (35)	t311 (14), ND (21)
C	94	30	IV (94)	t019 (42), t342(1), t975(2), t021(1), ND (48)
Others	17	ND	IV (7), V (1), NT (9)	t019 (6), t311 (1), ND (10)

Numbers within parenthesis indicate the number of isolates belonging to each *spa* type or SCC*mec* type.

ST denotes sequence type; ND, not determined; NT, non-typeable.

* A representative proportion of isolates from each pulse field type were studied.

Approximately half of study patients received prior antibiotic therapy for their SSSIs (Table 4). The most commonly used prior antibiotics were first generation cephalosporins followed by amoxicillin-clavulanate. Prior antibiotic therapy was more common among patients infected with CA-MRSA than in those without CA-MRSA (58,3% vs. 43%; p = 0,01). Prior antibiotic therapy received in the majority of patients with CA-MRSA was deemed to be inadequate based on *in vitro* susceptibilities (78%). Almost all patients with prior therapy (with or without CA-MRSA) required continued antibiotic therapy after the first study visit and in 82% of cases such therapy was adjusted to a different antibiotic. The most frequently used antibiotics after the first study visit were trimethoprim-sulfamethoxazole and clindamycin. Antibiotic therapy had a median duration of 10 days. Slightly more than half of the patients underwent drainage at the beginning of the study. There were no differences between patients infected with CA-MRSA and those who were not infected with CA-MRSA in terms of number, type of drainage (surgical vs. non-surgical) or length of antibiotic treatment. Patients with CA-MRSA (compared to those without CA-MRSA) were more commonly changed antibiotic therapy after the first study visit based on culture results (23,9% vs. 12,9%; p = 0.03).

**Table 4:** Most common antibiotic treatments, changes in therapy and drainage in patients with skin and skin structure infections.

Variables		Total N = 311n/N (%)	Community-associated MRSA N = 218n/N (%)	No Community-associated MRSA N = 93n/N (%)	p*
**Prior antibiotic treatment (before study visits)**	Total	167/311 (53,7%)	127/218 (58,3%)	40/93 (43,0%)	0,01
	Cephalosporin (1^st^ generation)	98/311 (31,5%)	76/218 (34,9%)	22/93 (23,7%)	0,05
	Amoxicillin/clavulanate	35/311 (11,3%)	24/218 (11%)	11/93 (11,8%)	0,83
	Trimethoprim-Sulfamethoxazole	17/311 (5,5%)	13/218 (6,0%)	4/93 (4,3%)	0,79
	Amoxicillin	13/311 (4,2%)	9/218 (4,1%)	4/93 (4,3%)	1,00
	≥2 antibiotics	25/311 (8,0%)	18/218 (8,3%)	7/93 (7,5%)	0,83
**Actual antibiotic treatment (at 1^st^ study visit)**	Total	309/311 (99,4%)	216/218 (99,1%)	93/93 (100%)	1,00
	Different from prior therapy	144/308 (46,8%)	109/217 (50,2%)	35/91 (38,5%)	0,06
	Parenteral therapy	87/304 (28,6%)	52/214 (24,3%)	35/90 (38,9%)	0,01
	Oral therapy	217/304 (71,4%)	162/214 (75,7%)	55/90 (61,1%)	–
**Actual antibiotic treatment, type of agent (at 1^st^ study visit)**	Trimethoprim-Sulfamethoxazole	137/311 (44,1%)	110/218 (50,5%)	27/93 (29,0%)	–
	Clindamycin	111/311 (35,7%)	78/218 (35,8%)	33/93 (35,5%)	–
	Cephalosporin (1^st^ generation)	35/311 (11,3%)	13/218 (6,0%)	22/93(23,7%)	–
	Quinolone	35/311 (11,3%)	24/218 (11,0%)	11/93 (11,8%)	–
	Rifampin	19/311 (6,1%)	15/218 (6,9%)	4/93 (4,3%)	–
	≥2 antibiotics	79/311 (25,4%)	54/218 (24,8%)	25/93 (26,9%)	0,70
**Antibiotic modification (after 1^st^ visit)**	Total	74/311 (23,8%)	58/218 (26,6%)	16/93 (17,2%)	0,07
	Based on cultures	64/311 (20,6%)	52/218 (23,9%)	12/93 (12,9%)	0,03
	Based on clinical outcomes	27/311 (8,7%)	21/218 (9,6%)	6/93 (6,5%)	0,36
**Length of antibiotic therapy in days, median (interquartile range)**		10 (7, 14)	10 (7, 14)	10 (7, 14)	0,56
**Drainage**	Total	166/311 (53,4%)	123/218 (56,4%)	43/93 (46,2%)	0,1
	Surgical drainage	82/311 (26,4%)	57/218 (26,2%)	25/93 (26,9%)	0,89
	Non-surgical drainage†	84/311 (27,0%)	66/218 (30,2%)	18/93 (19,3%)	–

MRSA denotes methicillin-resistant *Staphylococcus aureus*.

* Comparing patients infected with community-associated MRSA vs. those patients without community-associated MRSA.

† It refers to drainage without incision (e.g. needle drainage).

Patients infected with CA-MRSA were less commonly admitted to hospital than those without CA-MRSA (33,5% vs. 50,5%; p = 0,005). Overall cure rates were above 85%. There was no difference in cure rates between patients with CA-MRSA and those without CA-MRSA (Table 5). In the multivariable analysis two factors were independently associated with CA-MRSA in patients with SSSIs: presence of purulent lesions (OR 3,29; 95% confidence intervals 1.67, 6.49) and age <50 years old (OR 2,39; 95% confidence intervals 1.22, 4.70) (model c-index 0,75) (Table 6).

**Table 5:** Clinical outcomes in patients with skin and skin structure infections.

Clinical outcomes	Totaln/N (%)	Community-associated MRSAn/N (%)	No-Community-associated MRSAn/N (%)	p*
Hospitalization	120/311 (38,6%)	73/218 (33,5%)	47/93 (50,5%)	0,005
Surgical drainage after 1^st^ study consult	58/311 (18,7%)	37/218 (17,0%)	21/93 (22,6%)	0,25
Cure†	262/301 (87,0%)	184/210 (89,0%)	78/91 (85,7%)	0,65
Failure	8/301 (2,7%)	3/210 (1,4%)	5/91 (5,5%)	–
Indeterminate	31/301 (10,3%)	23/210 (11,0%)	8/91 (8,8%)	–
Death	5/311 (1,6%)	2/218 (0,9%)	3/93 (3,2%)	0,16

MRSA denotes methicillin-resistant *Staphylococcus aureus*.

* Comparing patients infected with community-associated MRSA vs. those patients without community-associated MRSA.

† 10 patients were excluded because lost of follow up; patients with indeterminate response were included in the denominator.

**Table 6:** Logistic regression model identifying clinical variables associated with community-associated MRSA in patients with skin and skin structure infections.

Variable	OR	95%CI	p
Purulent lesion	3.29	1.67, 6.49	0.0006
Multiple lesions	1.48	0.69, 3.17	0.32
Necrotic lesion	0.71	0.22, 2.31	0.57
Immunosuppression	1.17	0.34, 4.06	0.81
History of furunculosis	1.83	0.94, 3.56	0.07
Age <50 years	2.39	1.22, 4.70	0.01
Body mass index ≤25	1.55	0.83, 2.90	0.17

MRSA denotes methicillin-resistant *Staphylococcus aureus*; OR, odds ratio; 95%CI, confidence intervals 95%.

## Discussion

This prospective, multicenter study of CA-MRSA strains in adolescent and adult patients with SSSI in Argentina provided several findings.

Firstly, CA-MRSA has become the most common cause of skin and soft tissue infections in our patient without healthcare contact. From the total of 311 patients enrolled with SSTI 70% had CA-MRSA. The high rates of CA-MRSA observed among our patients with SSSI were similar to the rates observed in certain regions of the US [1], [8], [16]. This finding confirms our individual observations and will have significant impact on our antibiotic choices in the treatment of such patients. The occurrence of CA-MRSA as the predominant pathogen prompts an urgent change in the empirical therapy used to treat our community patients with SSSI. In addition, results from this study should encourage physicians to obtain cultures in patients with SSSI, most importantly in those areas where the prevalence of CA-MRSA or its antibiotic susceptibility are unknown [17].

Second, , this study reveals the predominance of the clone ST-30 SCC*mec* IV, PVL+ spa t019 among our patients from the community. This clone which was previously considered as an uncommon clone in Argentina appears to have displaced the previously predominant clone (ST5, SCC*mec* IV, PVL+) [6], [14]. Consistent with this finding we have recently described predominance of ST-30 SCC*mec* IV, PVL+ spa t019 among patients with invasive CA-MRSA infections in Argentina [18]. Predominance of one clone over the others could be associated with fitness advantages and reflects the dynamism of CA-MRSA epidemic [7], [19], [20]. Interestingly strains belonging to clonal complex 30 seem to have a different repertoire of enterotoxins, adhesins as well as important association with infective endocarditis [21]. Although our study was focused on community patients with SSSI CA-MRSA is a well known cause of invasive and hospital acquired infections [22]–[24] which are also occurring in our country [18], [25].

Third, SSSI caused by CA-MRSA have some characteristics that can be easily detected at the initial clinical evaluation. In our community population CA-MRSA is more common in patients <50 years old and presenting with purulent lesion such as abscesses or furuncles. Although PVL is not a primary determinant of outcome in patients with SSTI caused by CA-MRSA [26], [27] the leucocidin is associated with pus formation [28]. In fact our multivariable analysis showed that purulent lesions and age <50 years old were both independently associated with CA-MRSA. Also the frequent description of contacts with similar lesions emphases the important finding of a high degree of spread within closed groups [29], [30]. Cure rates were high among our patients. Importantly, clinical outcomes of patients with SSSI due to CA-MRSA were not different when compared to the clinical outcomes of patients without MRSA. These observations are in agreement with the literature indicating that SSSI due to CA-MRSA (usually PVL+) have good prognosis, and have similar outcomes to infections produced by PVL− MRSA [27] or MSSA [31].

Our study has several important limitations. Inclusion of patients may be biased by physicians who included those patients for whom they suspected CA-MRSA. We believe that the prospective nature of the study should have attenuated such potential bias. In fact these high rates of CA-MRSA were described in other SSSI studies as mentioned before [1], [8]. Second, antibiotic susceptibilities for CA-MRSA were not confirmed in our reference laboratory. However, susceptibility methods used by each microbiology laboratory were usually standard for most determinations. In addition, clinical decisions are based on such reports making them valuable to the study objectives. We did not have vancomycin minimum inhibitory concentration values for all strains. Therefore, we did not report such susceptibility in the current study. Is still worthy to mention that all isolates were reported susceptible to vancomycin by the different methods used (data not shown) at each laboratory. Last, the findings of this study are mostly limited to Buenos Aires city and state as well as the city of Santa Fe (Santa Fe state).

Despite the limitations described the current investigation indicates that CA-MRSA has become the most common cause of SSSI in our patient population. CA-MRSA is primarily seen in young adults presenting with purulent lesions and usually responds well to drainage plus antibacterial therapy. The clone ST30, SCC*mec* IV, PVL+, is now the predominant clone in adult patients suffering from SSSI in Buenos Aires, Argentina.
